# The impact of stress associated with caring for patients with COVID‐19 on career decisions, resilience, and perceived self‐efficacy in newly hired nurses in Jordan: A cross‐sectional study

**DOI:** 10.1002/hsr2.899

**Published:** 2022-10-25

**Authors:** Lourance A. E. Al Hadid, Marwa A. Al Barmawi, Rafi Alnjadat, Lo'ai Al Farajat

**Affiliations:** ^1^ Faculty of Nursing Al Balqa' Applied University Salt Jordan; ^2^ Nursing Department Alzaytoonah University of Jordan (ZUJ) Amman Jordan; ^3^ Irbid University College Al Balqa' Applied University Salt Jordan

**Keywords:** career decision, COVID‐19, nurses, resilience, stress

## Abstract

**Background and Aims:**

The decision to stay in nursing has been challenged by the recent coronavirus disease 2019 (COVID‐19) pandemic. New nurses joined the workforce and provided care to patients with COVID‐19 although they received limited training, which could have influenced their intention to stay in nursing. We aimed in this study to examine the impact of caring for patients with COVID‐19 on career decisions, resilience, and perceived self‐efficacy among newly hired nurses in Jordan. It also tested the predictors of intentions to stay among new nurses.

**Methods:**

This cross‐sectional quantitative study was conducted using an online electronic questionnaire form. The sample included newly hired nurses (*n* = 300) working in public hospitals and providing care to patients with COVID‐19 in different levels of acuity units. The perceived stress scale and Connor−Davidson resilience scale 25 were used to measure stress and resilience among nurses.

**Results:**

The majority chose nursing as their career, but they were not satisfied with the current work conditions or autonomy in decision‐making. Many nurses reported having moderate to high work‐related stress and low to moderate resilience. Among all variables in this study, financial income predicted mild intention to stay in nursing.

**Conclusions:**

Nurses expressed the presence of work‐related stress and low to moderate levels of resilience. As new nurses, exposure to these stress levels might lead to burnout. Nursing managers should take necessary measures to promote better work conditions and improve resilience to avoid nurses leaving the profession at times when there is a shortage.

## 1 INTRODUCTION

The decision to stay in nursing has been challenged by the recent coronavirus disease 2019 (COVID‐19) pandemic. The long‐standing shortage in the nursing workforce has been under pressure and many new, inexperienced nurses were forced to join fieldwork after receiving limited training.^1^ However, as exposure to COVID‐19 carried substantial morbidity and mortality rates among healthcare workers, some newly hired nurses were exposed to severely stressful situations.^2^, ^3^ Nurses caring for various levels of acuity among patients with COVID‐19 have been reported to experience depression and psychological trauma.^4^, ^5^ The continuous exposure to the significant risk of contracting the infection led to an increased magnitude of stress among nurses, especially those at the beginning of their careers.^6^, ^7^

The burden associated with COVID‐19 on healthcare systems was accompanied by limitations in nursing graduates' education, mentoring, and on‐job training, and those with limited nursing experience usually performed as newly hired nurses.^8^ These limitations might have influenced newly hired nurses' reflection on their careers, especially as information about mortality among healthcare workers is widely dispersed among all workers in the health field. As the pandemic still hits many countries with new waves of infection, newly graduated nurses are becoming more aware of the unprecedented challenges posed by the necessity of encountering this pandemic.^9^ These stressful conditions could influence nurses' decisions toward their nursing careers and the perceived self‐efficacy caused by the high mortality and morbidity rates associated with COVID‐19.^10^ It is therefore, necessary to examine both stress and self‐efficacy to better understand career decisions among newly hired nurses, especially those caring for patients with COVID‐19.

Newly hired nurses develop resilience as they train and practice nursing.^11^ However, the development of resilience could be questionable among the newly hired during the pandemic.^12^ Resilience has been recognized as a buffer against the impact of stress, thus facilitating adaptation to difficulties.^13^ Although there are a lot of studies examining resilience in nursing,^14^, ^15^ studying resilience in nursing graduates remains limited, particularly during the COVID‐19 pandemic. Resilience among new graduates and those with limited experience is an important factor that might significantly influence decisions about a career in nursing.^11^ Resilience among nursing students is a contributing factor that facilitates success in education, as nurses struggled when they transitioned from dependent to independent practice,^16^ but its impact on career decisions is yet to be addressed.

In the middle of this global shortage of trained nurses, it is necessary to maintain the nursing workforce in numbers and quality in a position that would assist in passing the impact of the COVID‐19 pandemic and perhaps other possible future health challenges. Therefore, it is necessary to address newly hired nurses and identify their level of resilience, perceived self‐efficacy, and the impact of the COVID‐19 pandemic on their nursing career decisions.

## 2 AIMS

This study aimed to examine the impact of perceived stress associated with caring for patients with COVID‐19 on career decisions, resilience, and perceived self‐efficacy among newly hired nurses in Jordan. It also tested whether stress, resilience, and other factors could predict intentions to stay among new nurses caring for patients with COVID‐19.

## 3 METHODS

### 3.1 Design

This is a cross‐sectional quantitative study conducted using an online electronic questionnaire form. Participants were provided with a link to the study questionnaire.

### 3.2 Sample and recruitment

The study population was newly hired nurses working in public hospitals and providing care to patients with COVID‐19 at different levels of acuity. The total sample size calculated using a confidence interval (CI) of 95% and statistical power of 80% and *α* of 0.05 on the G power software was 212 nurses. A list of newly hired nurses was obtained from the Ministry of Health, which included the field hospitals where they worked (*n* = 446). These field hospitals are newly established hospitals built specifically to receive and provide care to patients with COVID‐19 at all levels of care to reduce the impact on the main hospitals. These hospitals have emergency departments, intermediate care units, and ICUs. Newly hired nurses were defined as nurses hired during the pandemic, who worked for at least 4 months in those field hospitals and were all employed previously in high acuity units. We accessed the nurses through field visits to the hospitals in all regions of Jordan. The study aims and procedures were explained to new nurses, and they provided the researchers with their mobile phone numbers or emails to gain access to the study link. The survey pages allowed participants to move to the next page only if they have completed the previous page first. The timing was not restricted, but the number of attempts was restricted to “One Only.” Data were collected between June and August 2021.

### 3.3 Instruments

This section explains the instruments used in the study. It asked non‐identifying information about the participants and questions related to the topic of the study.

#### 3.3.1 Demographic characteristics

The demographic characteristics section included personal characteristics (i.e., gender, age, academic degree, other non‐nursing‐related degrees, work experience, and marital status). In addition, the demographic characteristics section comprised statements that described how nurses chose nursing as their career, their experience with nursing, and their plan to stay, such as “I chose nursing as my favorite career,” “The current situation in practice met my expectations concerning nursing career,” “My independence in decisions during work enhances my intention to stay at the profession,” “I will stay in nursing as the financial return is acceptable,” “I was trained adequately to perform the tasks of my current work,” and “Nursing is a good career.” A binary scale of agree or disagree was used to respond to the sentences. These sentences were based on the concepts explained in the Social Cognitive Career Theory (SCCT).^17^ The SCCT theory advocates that career development and selection are influenced by several factors, including financial considerations, internal and external environmental factors, educational and career achievements, and personal goals. Therefore, a person's career development can be impacted by single or multiple factors.^18^ The Arabic version of the statements was used in this study.

#### 3.3.2 The perceived stress scale (PSS)

The PSS has been adopted in this study to measure the level of stress.^19^ Questions in the PSS asked about the feelings and thoughts of an individual during the last month. PSS scores were obtained by reversing responses (e.g., 0 = 4, 1 = 3, 2 = 2, 3 = 1, and 4 = 0) to the four positively stated items (items 4, 5, 7, and 8), and then summing all scale items. The Arabic version adopted in this study has been tested and validated in several studies.^20^, ^21^

#### 3.3.3 Connor−Davidson resilience scale 25 (CD‐RISC‐25)

The CD‐RISC‐25 scale measured stress‐coping in individuals and aimed to treat stress and depression.^22^ It had 25 items that were rated between 0 and 4. The score of the scale was based on summing all items to provide a value range of 0 and 100 where higher scores reflected greater resilience. Based on the authors' recommendations, it was preferable not to consider independent factors or subscales for the RISC 25.^22^ Instead, the scale was measured using quartiles (i.e., Q1, Q2, Q3, Q4). Q1 described the score range for the lowest group, which reflected the least resilient, Q2 and Q3 the intermediate resilient, and Q4 described the most resilient group. The Arabic version of the CD‐RISC‐25 mean score for nurses was 60.08 ± 13.90,^14^ and the United States standard average was 82.^22^ The Cronbach's *α* of the CD‐RISC Arabic version, when used in nurses, was 0.94.^14^ The Arabic version was used in this study.

### 3.4 Ethical considerations

Permission was obtained from the Ethics Committee at the Ministry of Health. Participation was voluntary, and confidentiality and anonymity were ensured by not including any identifying information. Results were also reported without any remarks on the identity of nurses.

### 3.5 Statistical analysis

Both descriptive and inferential statistics were used to describe the study sample, its characteristics, and how these characteristics influenced the decision of future careers among nurses using SPSS version 26 (SPSS @IBM). The study team measured the levels of stress and resilience among nurses and how these influenced their decision to leave or stay in their careers. In addition, predictors of career decisions were measured to make future recommendations to enhance retention in the nursing workforce. The multiple linear regression model used in this study included workplace stress, resilience, financial income, degree of congruence between nurses' expectations of their career and the reality, independence of decisions, adequacy of training for nurses to provide care to patients, and perception of nursing as a good career on nurses' intentions to stay in nursing. Cronbach's *α* was used to test the internal consistency and reliability of the research instruments, as it is a suitable test for Likert's scale instruments.

## 4 RESULTS

This study included 300 participants (53.3%, *n* = 160 female nurses), who completed the study questionnaire (Table 1). Among these participants, 50.7% (*n* = 152) were single, and most of them had baccalaureate degrees (82.0%, *n* = 246) as their highest academic degree in nursing. Many nurses were new graduates with experience of fewer than 2 years (70%, *n* = 210), and maximum years of experience of fewer than 6 years. Those were newcomers to Jordan who had previously worked in other countries. All participants provided care to patients with COVID‐19 for a period of 6−12 months and worked in the emergency department (58%, *n* = 174) and the ICU (42%, *n* = 126); nurses provided care only to patients with COVID‐19 during this period. None of the nurses received previous training in caring for acute cases during pandemics.

**Table 1:** Personal characteristics and beliefs about a nursing career (*n* = 300)

Factor	Category	*n*	%
Age range 20−40 (average 28.34, SD 4.77)				
Gender	Male	140	46.7
	Female	160	53.3
Marital status	Single	152	50.7
	Married	148	49.3
Academic degree	Diploma	20	6.7
	Baccalaureate	246	82.0
	Masters	34	11.3
Years of experience	1−3	210	70
	4−6	90	30
Area of work	Emergency department	174	58
	ICU	126	42
I chose nursing as my favorite career.	Disagree	83	27.7
	Agree	217	72.3
The current situation in practice met my expectations concerning a nursing career.	Disagree	202	67.3
	Agree	98	32.7
The independence of my decisions during work enhances my intention to stay in the profession.	Disagree	215	71.7
	Agree	85	28.3
I will stay in nursing as the financial return is acceptable.	Disagree	62	20.7
	Agree	238	79.3
I was trained adequately to perform the tasks of my current work.	Disagree	150	50.0
	Agree	150	50.0
Nursing is a good career.	Disagree	118	39.3
	Agree	182	60.7

The results revealed that almost two‐thirds of the participants (72.3%, *n* = 217) chose nursing as their career willingly. It is worth mentioning that in Jordan, it is the high school average that determines the school which any student can join. Therefore, sometimes some students cannot go to the schools of their choice. When asked whether the current work conditions met their expectations of a nursing career, 67.3% (*n* = 202) responded negatively. Many participants (71.7%, *n* = 212) also responded that they did not have independence in decisions at work, which in turn discouraged their intention to stay in the profession. While 79.3% (*n* = 238) said that will stay in nursing as the financial return is acceptable, 60.7% (*n* = 182) indicated that nursing is a good career. During their nursing work, 50% of the participants received adequate training on skills required in their current workplace.

The Cronbach's *α* value for the PSS was 0.841 and the CD‐RISC‐15 0.930, indicating acceptable reliability and internal consistency values. The skewness and kurtosis values of both instruments showed a distribution of values that did not violate normal distribution principles (Table 2). The total mean score of the participants on the PSS was 21.12 (SD 1.99), and more than half of the participants reported having moderate stress levels (55%, *n* = 165). The mean score on the CD‐RISC‐15 was 74.98 (14.01) and the low resilience group (Q1) was 23% (*n* = 69), while the moderate to high averages (Q3 and Q4) represented 52% (*n* = 156) of the participants.

**Table 2:** Workplace stress and resilience among new nurses caring for patients with COVID‐19

Scale	Mean (SD)	Range	Category	*n* (%)	Skewness (SE)	Kurtosis (SE)	*α* *
Perceived stress	21.12 (1.99)	16−27	Low stress (less than 14)	128 (42.7%)	0.270 (0.141)	0.442 (0.281)	0.841
			Moderate (14−26)	165 (55%)			
			High (27−40)	7 (2.3%)			
CD‐RISC	74.98 (14.01)	23−100	Q1	69	−0.845 (0.141)	0.901 (0.281)	0.930
			Q2	75			
			Q3	85			
			Q4	71			

Abbreviations: COVID‐19, coronavirus disease 2019; Q, quartile.

* Cronbach's *α*.

### 4.1 Predictors of intention to stay in nursing among nurses caring for patients with COVID‐19

In addition to workplace stress and resilience, we asked questions about the effect of financial income, degree of congruence between nurses' expectations of their career and the reality, independence of decisions, adequacy of training for nurses to provide care to patients, and perception of nursing as a good career on nurses' intentions to stay in their work (Table 3).

**Table 3:** Predictors of intentions to stay in Nursing among new nurses caring for patients with COVID‐19 (*n* = 300)^a^

Predictor^b^	*β*	*R*	*R* ^2^	*F* change	Sig
I will stay in nursing as the financial return is acceptable.	0.130	0.330	0.109	5.146	0.019
The current situation in practice met my expectations concerning a nursing career.	−0.035	0.035	0.001	0.372	0.543
The independence of my decisions during work enhances my intention to stay in the profession.	−0.33	0.048	0.002	0.318	0.573
Nursing is a good career.	0.38	0.145	0.021	0.448	0.504
Total perceived stress (PSS)	−0.073	0.073	0.005	0.005	0.207
Nurses' resilience (CD‐RISC‐25)	0.006	0.073	0.005	0.000	0.924

Abbreviations: CD‐RISC‐25, Connor−Davidson resilience scale 25; COVID‐19, coronavirus disease 2019; PSS, perceived stress scale.

^a^ Confidence interval 95%.

^b^ Dependent variable: I am planning to continue in my current work as a nurse.

Results of the regression indicated that there was a significant association between the financial income of working as a nurse and the intention to stay in nursing. The financial income had partially predicted the intention to stay in nursing by 10.9% (*t* = 2.268, *F* (1, 298) = 5.146, *p* = 0.019, *R* ^2^ = 0.109, CI 95%). The individual predictors were examined further and indicated no significant association between intention to stay in nursing, workplace stress, resilience, and all individual questions (refer to Table 1).

## 5 DISCUSSION

The impact of COVID‐19 on healthcare workers, including nurses, is still ongoing worldwide. We examined some of the factors associated with caring for patients with COVID‐19, as a serious health threat, to nurses with limited experience, and tested its impact on career decisions. Our findings indicated that most nurses experienced moderate to high workplace stress levels and just less than half of them had low to moderate resilience. These findings agree with previous studies where all nurses from the United States, Japan, and the Republic of Turkey had moderate resilience ratings.^23^ However, they received much public support and gratitude, which might have helped with resilience and compassion fulfillment.^23^

Nurses' resilience is a vital component of their emotional work of coping with patients' illnesses and death. A nurse, who lacks resilience, may burn out and decide to leave nursing entirely.^24^ Nurses, who tested positive for COVID‐19, had lesser resilience than those, who tested negative.^23^ In our sample, the acceptable financial reward was significantly associated with a decision to remain in the nursing profession.^25^ Our study confirmed the importance of a rewarding system and its association with nurses to stay in their nursing careers.^26^

A previous study indicated that students usually select nursing for social and cultural expectations, which is a long‐term investment.^27^ This study also indicates that most nurses willingly selected nursing as their career. In addition, many nurses reported dissatisfaction with the level of independence and level of participation in the decision‐making process. They reported that they intend to stay in the profession and work as nurses due to financial income. The roles and professional status of nurses have increased over the past few decades; however, they might still believe that they have many limitations in the decision‐making process concerning how work is performed and how care is planned for their patients.^28^ In addition, half of the nurses reported being prepared to care for patients with COVID‐19. However, this result did not significantly influence their intention to stay in nursing. Heavy workloads, lack of resources, burnout, and stress associated with the pandemic are the driving reasons for the increased number of nurses quitting the field.^24^ Our data show that nursing professionals, who cared for COVID‐19 patients, expressed their intention to leave the profession. These elements, we believe, constitute extra employment pressure that enhances nurses' intent to leave.

The SCCT advocates that persons' behaviors are not only a result and outcome of individual behaviors and selections. These behaviors are usually influenced by financial considerations, internal and external environmental factors, educational and career achievements, and personal goals.^17^ Nurses' intention to stay in nursing is influenced by their financial income,^29^ especially as the unemployment rates, have surged during and post‐COVID‐19 periods due to many reasons, including lockdowns and employee layoff rates in different businesses.^30^ Individuals' decisions can then be changed due to various situations and personal developments. The SCCT provides theoretical support to explain the reasons and motivations for intentions to stay in nursing.^17^

Workplace stress was significant among nurses, who reported moderate to high‐stress levels. Therefore, the decision to stay or leave the profession among nurses was a serious concern to healthcare planners during the COVID‐19 pandemic.^31^ Nurse turnover is not only costly but also difficult to replace, which could influence the quality of care.^32^

Approximately half of the nurses in this study experienced low resilience levels. Resilience must be emphasized among nurses, particularly junior nurses, to improve retention, productivity, and control over workplace stress and burnout.^10^, ^15^ The impact of low resilience on new nurses could be serious as it has been associated with higher stress, burnout, and compassion fatigue.^33^ Building resilience among nurses is necessary to promote self‐care and reduce compassion fatigue. Nurses' job satisfaction and retention, as well as the quality of care offered in their units, may benefit from resilience‐promoting interventions.^34^

Limitations of this study include using an online questionnaire as no control can be emphasized on who, when, and where the questionnaire was completed. In addition, there was a limited control on sampling bias; only nurses interested in completing the study filled out the questionnaire. Completing a quantitative questionnaire limits nurses' ability to comment or add to the responses and reflect further on their personal experiences and thoughts. In addition, there was no comparison group to compare levels of resilience against, which makes it difficult to make conclusions about the sample of this study.

## 6 CONCLUSION

Data from this current study indicated that most newly hired nurses reported low to moderate levels of occupational stress, and only around half of them showed low to moderate resilience. In addition, the majority picked nursing as their preferred job, expressing a high level of autonomy in decision‐making as well as existing work conditions that were unfavorable and as a result affected their decision to continue in nursing. Only financial return was a significant predictor to stay in nursing among nurses in this study. As such, examining the influence of caring for COVID‐19 patients on newly hired nurses' career decisions, resilience, and perceived self‐efficacy is becoming an important focus for the management and hospital administrations. An additional area of assessment for future studies could also include an interventional program. The assessment could help determine the potential of the intervention to sustain or continue to positively impact the lives of junior nurses in Jordan.

## 7 IMPLICATIONS FOR NURSING PRACTICE

The COVID‐19 pandemic has transformed the healthcare trajectory worldwide. The advent of the crisis has far‐reaching implications in healthcare that go beyond the disease. New nurses experienced work stress and low to moderate resilience, which could be associated with decisions to leave nursing. In addition, high stress and low resilience have been reported to cause an increased negative impact on productivity, loyalty, and even patient safety. Therefore, we recommend the provision of counseling services to nurses, especially the newly hired, who are still fresh in their careers and whose coping strategies are not yet well‐established. We recommend establishing an effective communication system that ensures confidentiality and seriousness when handling complaints of all nurses. We also recommend that stakeholders in healthcare take measures to promote better work conditions and improve resilience to avoid nurses' burnout and improve retention of nurses by assessing nurses' needs, increasing teamwork, and improving communication at the workplace.

## AUTHOR CONTRIBUTIONS

**Lourance A. E. Al Hadid**: conceptualization; data curation; formal analysis; investigation; methodology; project administration; resources; supervision; writing – original draft; writing – review & editing. **Marwa A. Al Barmawi**: data curation; writing – review & editing. **Rafi Alnjadat**: data curation; investigation; resources; writing – original draft; writing – review & editing. **Lo'ai Al Farajat**: conceptualization; data curation; formal analysis; investigation; writing – original draft.

## CONFLICT OF INTEREST

The authors declare no conflict of interest.

## TRANSPARENCY STATEMENT

The lead author Lourance A. E. Al Hadid affirms that this manuscript is an honest, accurate, and transparent account of the study being reported; that no important aspects of the study have been omitted; and that any discrepancies from the study as planned (and, if relevant, registered) have been explained.

## Data Availability

The data sets used and/or analyzed during the current study are available from the corresponding author upon reasonable request. Lourance A. l.‐Hadid accepts full responsibility for the accuracy and integrity of the data provided.
